# Retracted: Stearoyl-CoA Desaturase 1 Potentiates Hypoxic plus Nutrient-Deprived Pancreatic Cancer Cell Ferroptosis Resistance

**DOI:** 10.1155/2022/9809570

**Published:** 2022-02-23

**Authors:** Oxidative Medicine and Cellular Longevity

*Oxidative Medicine and Cellular Longevity* has retracted the article titled “Stearoyl-CoA Desaturase 1 Potentiates Hypoxic plus Nutrient-Deprived Pancreatic Cancer Cell Ferroptosis Resistance” [1] due to concerns with figure duplications in Figure 1(b) as originally noted on PubPeer [2]. Specifically, the DMSO Nor panel is duplicated with the DMSO H/NS panel, and the Erastin Nor+Fer-1 panel is duplicated with the SAS Nor+Fer-1 panel. In both cases, the field of view is different between the panels. The author responded to explain that the duplications were introduced due to an error when preparing their manuscript, however, this did not satisfy the concerns of the editorial board and the article is retracted due to concerns with the reliability of the data.

The authors do not agree to the retraction.
